# The prevalence of gingivitis and related risk factors in schoolchildren aged 6–12 years old

**DOI:** 10.1186/s12903-022-02670-9

**Published:** 2022-12-21

**Authors:** Xiaoyu Liu, Jianhui Xu, Siwei Li, Xueqin Wang, Jin Liu, Xin Li

**Affiliations:** The Second Affiliated Hospital of Jinzhou Medical University, Jinzhou, China

**Keywords:** Gingivitis, Calculus, Children, Oral prevention, Risk factors

## Abstract

**Background:**

According to epidemiological studies, gingivitis is a common disease. However, its morbidity, considerably varies among individual. This study aimed to investigate the epidemiological characteristics of gingivitis, including prevalence, severity, intraoral distribution, and associated risk factors, in children aged 6–12 years in Jinzhou, China.

**Methods:**

A multistage, whole-group, randomized sample of 2880 children aged 6–12 years in Jinzhou City, China, was selected and clinically examined. Each selected child completed a questionnaire on sociodemographic factors and oral health behaviors in cooperation with the investigator and teacher. Gingival bleeding refers to the bleeding of 10% or more teeth under the condition of ingivitis. Gingivitis was further categorized into localized gingivitis (30% ≥ number of teeth positive for gingival bleeding ≥ 10%) and generalized gingivitis (number of positive for gingival bleeding > 30%). The score of gingival bleeding was recorded using the Gingival Index.

**Results:**

The prevalence of gingivitis in children aged 6–12 years in Jinzhou was 28.58%, including 701 cases of localized gingivitis (24.3%) and 122 cases of generalized gingivitis (4.2%). There were 429 cases (28.3%) of gingivitis in males and 394 cases (28.9%) in females, with no statistically significant difference in prevalence between males and females (*P* > 0.05). Chi-square tests and binary logistic regression analysis showed that aging, dental calculus, plaque, and dental crowding were significantly associated with a high prevalence of gingivitis.

**Conclusions:**

Our study showed that dental calculus, large amount dental plaque, poor oral health behavior, and oral health awareness are associated with the prevalence of gingivitis and maintaining children's oral health requires professional guidance and regular preventive care.

## Background

Chronic gingivitis is the most common periodontal infection in children and adolescents and includes chronic gingivitis caused by plaque, steroid hormone-related gingivitis, and drug-influenced gingival overgrowth, and the most common is chronic gingivitis [1]. Plaque microorganisms can initiate periodontal diseases [2]. Gingivitis is of particular clinical significance as it is considered a pre-existing stage of periodontitis, the development of which occurs in only long-term gingivitis [3]. The initial symptoms of gingivitis are not obvious, and bleeding on probing and redness of the gums do not occur until early lesions of gingivitis are present. It is usually painless, rarely causes spontaneous bleeding, and its clinical symptoms are not obvious enough for most patients to recognize the disease [4]. A study showed that during adolescence, periodontal tissues become more sensitive to several irritants, such as plaque, calculus, and food debris collected in the gingival sulcus, owing to an increase in sex hormones (estrogen and progesterone) [5]. It is therefore necessary to systematically examine children's oral hygiene and educate them about oral hygiene and preventive measures of oral diseases before they reach puberty. Several studies have shown that effective long-term prevention of gingivitis can control the onset of attachment loss, making the long-term prevention essential for the primary prevention of periodontitis [3, 6]. As mentioned above, maintaining good and healthy oral hygiene environment during childhood is beneficial for oral health in adulthood. In addition to a plaque, other factors including body mass index (BMI), gender, and parental education may directly or indirectly influence the development of gingivitis [7]. A survey in Sichuan province, China showed that children from urban households had a lower prevalence of gingivitis compared to that had by children from rural households and that higher levels of parenting had a positive impact on gingival status [8]. 

Although epidemiological studies have consistently shown that gingivitis is a common disease [9], there is considerable variation between prevalence rates, which can be explained by inter-population variability; however, the differences may also be due to the varying criteria used for diagnosis. Numerous epidemiological studies evaluate gingivitis based on the community periodontal index (CPITN) [10]. However, the CPITN is not used to define the gingivitis grades. The CPITN were used to screen for periodontitis; therefore, the severity of the disease could not be defined. The joint EFP/AAP workshop defined gingivitis as children with a positive gingival bleeding score of ≥ 10% no attachment loss or periodontal probing depth (≥ 4 mm), which in turn was categorized into localized and generalized gingivitis. Patients were considered to have healthy periodontal tissue if they presented with a positive gingival bleeding score of < 10% at probing and if the periodontal tissue was intact [11].

According to the results of an oral epidemiological survey, prevalence varies greatly from region to region [8, 12–15]. The prevalence of gingivitis in children aged 12 years was 46.63% in the southern Chinese province of Sichuan [8] and 29.1% in the central province of Shandong [12]. The prevalence also varies by ethnic group, with 93%, 61%, and 88% prevalence reported among the Dai, Hani, and Lisu ethnic groups in Yunnan Province, China, respectively [13–15]. No data are available for Jinzhou, which is a transportation hub in the north with a complex population comprising multiple ethnic groups. Therefore, the purpose of this study was to investigate (1) the prevalence of gingivitis in children aged 6–12 years in Jinzhou, China, (2) risk factors associated with gingivitis, and (3) factors associated with the severity of gingivitis.

## Methods

A multi-stage, whole-group, random sampling method was used. Based on the Fourth National Oral Epidemiological Survey, the prevalence of gingivitis among 12-year-old children in Jinzhou, China is approximately 58% [9]. The sample size was increased by 10%, considering issues such as missed visits and individual samples that did not meet the inclusion criteria. A total of 2880 children aged 6–12 years were sampled from all counties and districts in Jinzhou, China. The specific demographic characteristics are presented in Table 1. This epidemiological investigation was approved by the Ethics Committee of the Second Hospital of Jinzhou Medical University, China. A signed informed consent form was obtained from the family of the child before the children participated in the study.

**Table 1:** Survey population composition

	N	%
*Gender*		
Male	1516	52.64
Female	1314	47.36
*Age(year)*		
6	359	12.47
7	416	14.44
8	322	11.18
9	583	20.24
10	333	11.56
11	416	14.44
12	451	15.66
*Area*		
Han	2004	69.58
Man	728	25.28
Other	148	5.14

Inclusion criteria: (1) Aged 6–12 years, (2) informed of the study details and with a consent form signed: (3) Living in Jinzhou City for more than 6 months, and (4) All the first molars have erupted completely.

Exclusion criteria: (1) Children with congenital oral diseases, (2) Children who were unable to cooperate with the examination after behavioral and psychological induction, (3) Children undergoing orthodontic treatment, and (4) children whose first molar had not yet erupted.

### Inspection methods

On-site inspections occurred between September 2021 and December 2021. Examination was performed in the sitting position under LED light. With Oral examination instruments under standard infection control conditions (disposable oral goggles, disposable oral probes, inspection gloves, metallic WHO CPI probe and medical cotton swabs).The WHO Oral Health Assessment Form, which is a modified version of clinical examination, was used to record clinical findings. Dental examination was conducted by six dental examiners, the oral cavity of each participant was assessed by a clinical examiner who was assisted by a recorder. The clinical examiner is a practicing dentist with at least 3 years of experience.

After the clinical examination, teachers uniformly distributed the questionnaires to parents. The questionnaire to the parents with reference to the 4th National Oral Epidemiological Survey. The questionnaire covered general information about the respondents, their oral health behavior, dietary habits, poor oral habits, and family oral health awareness. Quality checks were performed by an auditor after the questionnaire was returned.All examination and questionnaire results were inputted into an electronic computer to create a database for statistical processing and analysis of data, which was used to calculate the prevalence and risk factors of gingivitis, respectively, in children aged 6–12 years in Jinzhou, China.

### Oral examination

Using the GI as a screening standard for gingivitis (score 2 and 3 of the GI with bleeding on probing), a probe-based examination was combined with visual examination. The CPI probe was gently inserted into the gingival sulcus, and the entire area of the gingival sulcus was probed with the probe being parallel to the long axis of the tooth and close to the root. Short up and down quivering movements were then performed.Based on the feeling of dental calculus, it is recorded that there is a dental calculus found during exploration. In addition, we assessed for bleeding gums and scored the gum condition with not more than 20 g of force. The fraction of each tooth in the mouth, the non-eruption of permanent teeth, and the fraction of temporary teeth in the same position were recorded, and the second and third molars were not recorded. Gingivitis was defined as children with ≥ 10% positive teeth for gingival bleeding and with no attachment loss or periodontal probing depth (≥ 4 mm), which was further categorized into localized gingivitis (30%number of teeth positive for gingival bleeding ≥ 10%) and generalized gingivitis (number of positive teeth for gingival bleeding > 30%) [10]. No other diagnostic methods or equipment were used, including oral radiography. The Löe–Silness plaque index, scoring standard: 0 = Absence of plaque in the gingival margin area; 1 = Thin plaque on the tooth surface at the gingival margin area, but if the plaque can be scraped off with the side of the probe tip,it is not easy to see by visual inspection; 2 = Moderate amount of plaque visible on the gingival margin or adjacent surfaces; 3 = Large amounts of plaque in the gingival sulcus or gingival margin area and adjacent surfaces. Levels 0, 1, 2, 3 corresponds to the four levels: none, small amount, medium amount and large amount. Several risk factors that may affect gingivitis, including dental calculus, number of decayed fillings, BMI [16], deep overjet, and crowding, were also examined. In the early stages of the epidemiological investigation, all participants received unified clinical training. Reliability of examiners was assessed by reliability tests on 20 subjects prior to the final survey. Kappa values of the examiner’s own standard consistency test were 0.90, 0.92 and 0.87, respectively. Kappa value of the standard consistency test among examiners was 0.80.The feasibility of the questionnaire was tested and the oral examination terminology was unified. During the formal survey, 5% of all subjects examined per investigator per day were selected for repeat experiments, and the clinical findings of the other examiner were reviewed by the first of two examiners in the order of 1 and 2, 2 and 3, and 3 and 1.

### Statistical analysis

The data from this survey were double-entered using Epidata, and all statistical processing of the data was performed using SPSS 25.0. Percentages were used to describe categorical data, showing the prevalence of each factor. Two-factor analysis using Pearson’s chi-square test was performed, and statistically significant variables were further included in the binary logistic regression analysis and in the calculation of Odd ratio. (OR) and 95% confidence intervals (CI) to identify risk factors. The statistically significant level was α = 0.05, *and P* < 0.05 was considered statistically significant.

## Results

Of a total of 3100 participants, 15 did not complete the questionnaire, the parents of 15 participants did not sign the informed consent form, and 90 were excluded as they did not meet the inclusion criteria, resulting in 2880 participants receiving a complete clinical examination and completing the questionnaire (participation rate, 92.9%). Gingivitis was detected in 823 participants, with an overall prevalence of 28.58%. The prevalence of localized and generalized gingivitis was 24.34% and 4.24%, respectively. Of the 2,880 participants, 52.64% were males, and 47.36% were female. The prevalence of gingivitis in males was 28.3%, of which 23.28% was localized gingivitis and 5.01% was generalized gingivitis. The prevalence of gingivitis in females was 28.89%, of which 25.51% was localized gingivitis and 3.37% was generalized gingivitis. The prevalence of gingivitis tended to increase with age, and age had a statistically significant effect on the prevalence of gingivitis, with a statistically significant difference (*P* < 0.05). The prevalence of gingivitis was approximately the same among the different ethnic groups. In addition, there was no statistical difference in the prevalence of gingivitis between urban and rural populations (*P* > 0.05).

The chi-square test was used to analyze the relationship between demographic factors and gingivitis (Table 2). Age had a statistically significant effect on the prevalence of gingivitis, with a statistically significant difference (*P* < 0.05). Ethnicity did not affect the prevalence of gingivitis (*P* > 0.05).

**Table 2:** Relationship between demographics and prevalence of gingivitis in children aged 6–12 years in Jinzhou

Variable	*N*	Gingivitis			Normal (n = 2057)	X^2^	*P*
		Total (n = 823)	Localized gingivitis (n = 701)	Generalized Gingivitis (n = 122)			
		n (%)	n (%)	n (%)	n (%)		
*Gender*							
Male	1516	429 (28.3)	353 (23.28)	76 (5.01)	1087 (71.7)	0.121	0.728
Female	1364	394 (28.89)	348 (25.51)	46 (3.37)	970 (71.11)		
*Age*							
6	359	63 (17.55)	58 (16.16)	5 (1.39)	296 (82.45)	73.27	0.000
7	416	78 (18.75)	66 (15.86)	12 (2.89)	338 (81.25)		
8	322	102 (31.68)	91 (28.26)	11 (3.42)	220 (68.32)		
9	583	169 (28.99)	149 (25.56)	20 (3.43)	414 (71.01)		
10	333	99 (29.73)	81 (24.32)	18 (5.41)	234 (70.27)		
11	416	132 (31.73)	110 (26.44)	22 (5.29)	284 (68.27)		
12	451	180 (21.87)	146 (31.53)	34 (7.34)	271 (60.08)		
*Ethnicity*							
Han	2004	574 (28.64)	489 (24.4)	85 (4.24)	1430 (71.36)	1.481	0.477
Man	728	213 (29.27)	180 (24.73)	33 (4.53)	515 (70.74)		
Other	148	36 (24.32)	32 (21.62)	4 (2.7)	112 (75.68)		
*Area*							
Urban	2580	732 (28.37)	623 (24.15)	109 (4.22)	1848 (71.63)	0.506	0.477
Rural	300	91 (30.33)	78 (26)	13 (4.33)	209 (69.67)		

**Fig. 1:**
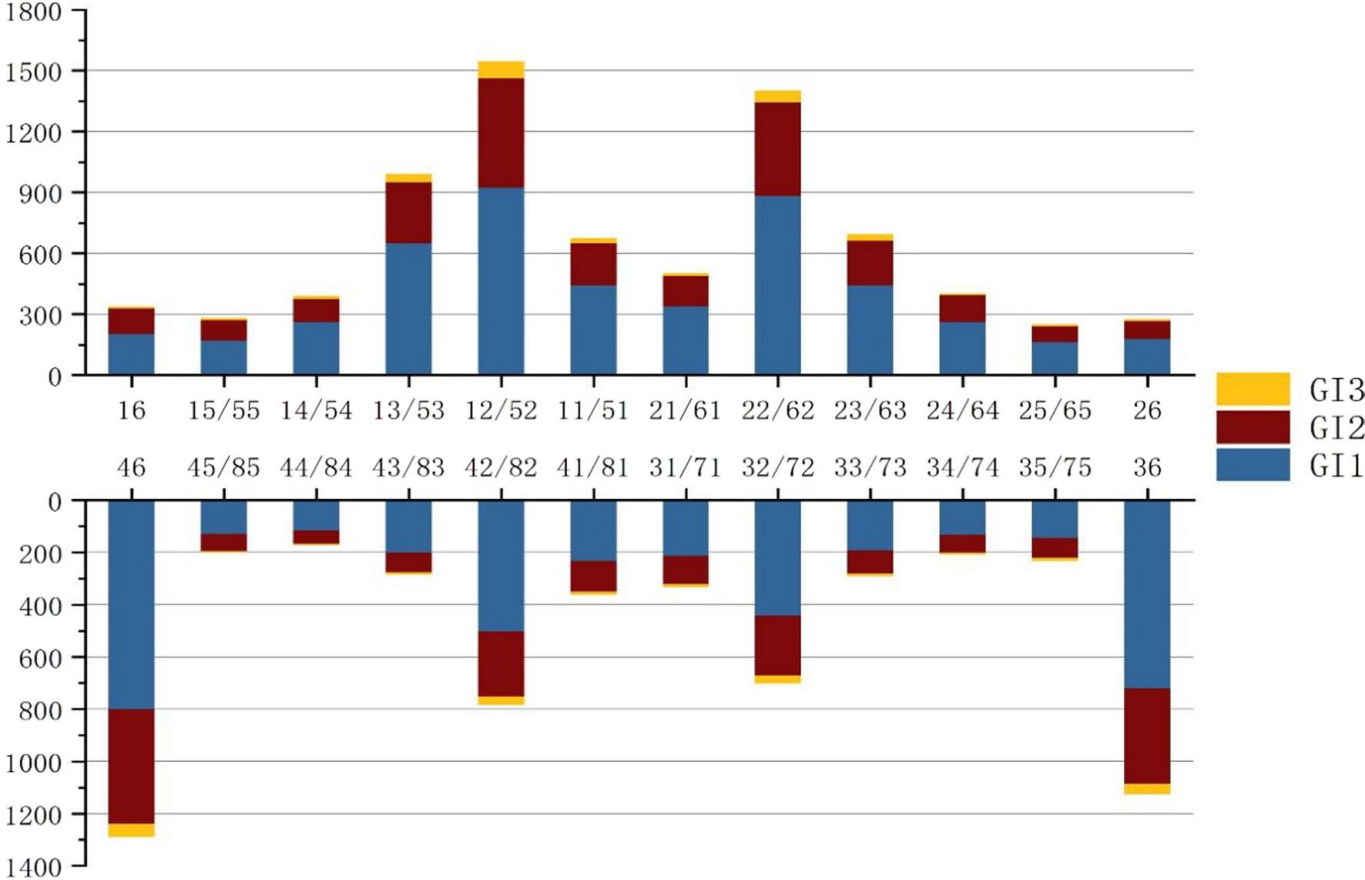
Distribution characteristics of gingival index scores for different tooth positions

Figure 1 shows the characteristics of the intraoral distribution of the GI score for each tooth position, with the upper right lateral incisor having the highest number of prevalent teeth in the total tooth position. The first lower right molar was the most affected lower jaw teeth. Overall, the anterior teeth scored higher on the GI than that scored by the posterior teeth, and the right side scored higher than that scored on the left.

Relationship between each risk factor and gingivitis and between oral health behaviors and gingivitis are reported in Table 3. Plaque, dental calculus, crowding, and deep overjet was significantly related with the prevalence of gingivitis (*P* < 0.05). There was no statistically significant relationship between BMI and the prevalence of gingivitis. Among oral health behaviors (Table 4), the prevalence of gingivitis was significantly affected by bleeding from brushing, time spent brushing, flossing, and rinsing after meals (*P* < 0.05).

**Table 3:** Analysis of risk factors associated with gingivitis in children aged 6–12 years in Jinzhou

Variable	*N*	Gingivitis (n = 823)	Normal (n = 2057)	X^2^	*P*
		n (%)	n (%)		
*Dental plaque*					
No	414	42 (10.15)	372 (89.85)	818.32	0.000
Small amount	1334	143 (10.72)	1191 (89.28)		
Medium amount	996	528 (53.01)	468 (46.99)		
Large amount	136	110 (80.88)	26 (19.12)		
*Dental calculus*					
No	1996	221 (11.07)	1775 (88.93)	976.199	0.000
Yes	884	602 (68.09)	282 (31.91)		
*BMI*					
Slim	104	33 (31.73)	71 (68.27)	0.528	0.913
Normal	1426	406 (28.47)	1020 (71.53)		
Obesity	824	234 (28.4)	590 (71.6)		
Overweight	526	150 (28.52)	376 (71.48)		
*Crowded teeth*					
No	2086	271 (12.99)	1815 (87.01)	900.434	0.000
Yes	794	552 (69.52)	242 (30.48)		
*Deep overjet*					
NO	2621	646 (23.5)	1975 (76.5)	220.467	0.000
Yes	259	177 (79.92)	82 (20.08)

**Table 4:** Relationship of oral health behaviors and health awareness with gingivitis

Variable	*N*	Gingivitis (N = 823)	Normal (N = 2057)	X^2^	*P*
		n (%)	n (%)		
*Bleeding from brushing teeth*					
Never	2038	400 (19.63)	1638 (80.37)	325.874	0.00
Sometimes	762	355 (46.58)	407 (53.41)		
Frequently	80	68 (85)	12 (15)		
*Daily brushing frequency*					
Three or more times a day	48	13 (27.08)	35 (72.92)	0.24	0.887
Twice a day	2124	603 (28.39)	1521 (71.61)		
Once a day	708	207 (29.24)	501 (70.76)		
*Brushing time*					
> 3 min	542	139 (25.65)	403 (74.35)	6.223	0.045
2 –3 min	1542	432 (28.02)	1110 (71.98)		
< 2 min	796	252 (31.66)	544 (68.34)		
*Flossed or not*					
Yes	1153	243 (21.08)	910 (78.92)	53.004	0.000
No	1727	580 (33.58)	1147 (66.42)		
*Whether to rinse your mouth after a meal*					
Every time	505	143 (28.32)	362 (71.68)	0.449	0.799
Sometimes	1876	543 (28.94)	1333 (71.06)		
No mouthwash	499	137 (27.45)	362 (72.55)		
*Have you seen a dentist in the past year*					
Yes	2007	445 (22.17)	1562 (77.83)	133.039	0.000
No	873	378 (43.3)	495 (56.7)		
*Brushing prevents bleeding gums*					
Yes	2068	595 (28.77)	1473 (71.23)	0.252	0.882
No	428	118 (27.57)	310 (72.43)		
Unknown	384	110 (28.65)	274 (71.35)		
*It is important to have regular dental check-ups*					
Yes	2820	807 (28.62)	2013 (72.38)	10.576	0.005
No	17	7 (41.18)	10 (58.82)		
Unknown	43	9 (20.93)	34 (79.07)		
*Causes of bleeding from brushing teeth*					
Normal	72	25 (34.72)	47 (65.28)	4.205	0.24
Excessive force	566	175 (30.92)	391 (69.08)		
Inflammation of the gums	1569	444 (28.3)	1125 (71.7)		
Unknown	673	179 (26.6)	494 (73.4)		
*Your parents will supervise your tooth brushing every day*					
Yes	1425	396 (27.79)	1029 (72.21)	1.241	0.538
No	232	72 (31.03)	160 (68.97)		
Sometimes	1223	355 (29.03)	868 (70.97)		

**Fig. 2:**
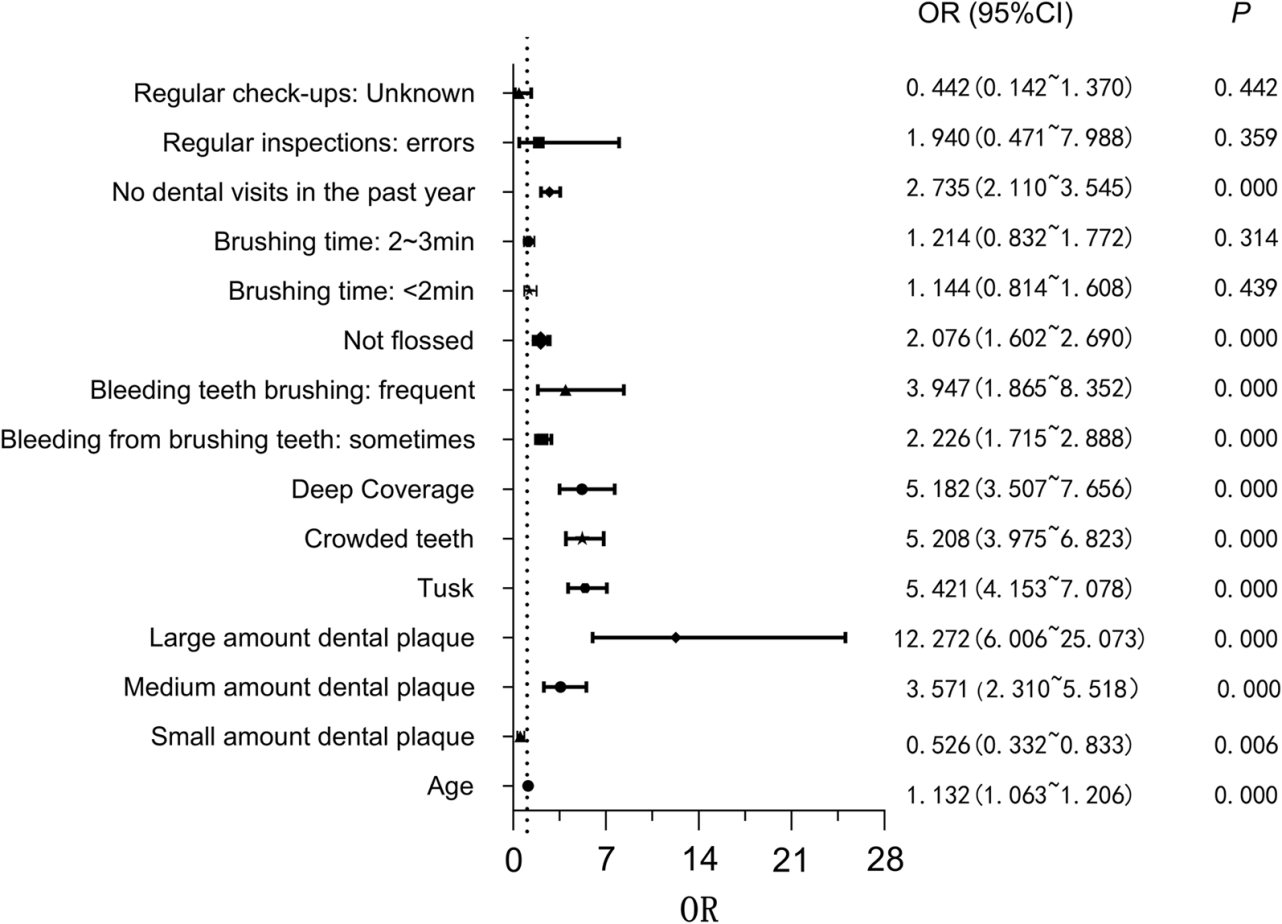
Binary logistic visual regression analysis of risk factors for gingivitis

Variables that were statistically significant (*P* < 0.05) in the chi-square test were included in the binary logistic regression analysis (Fig. 2). We used OR = 1 as the criterion for differentiating the risk factors. Age, dental calculus, medium amount/ large amount dental plaque, crowded teeth, deep overjet, bleeding during brushing that was frequent or occurred sometimes, lack of flossing, and the visiting a dentist in a year were associated with the development of gingivitis.

**Fig. 3:**
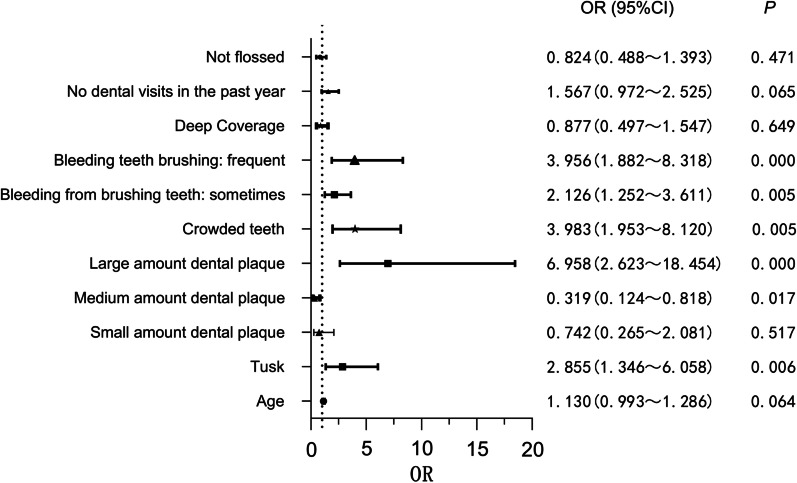
Binary logistic visual regression analysis of risk factors for localized versus generalized gingivitis

The risk factors associated with gingivitis were then included in a binary logistic regression analysis with localized versus generalized gingivitis as the dependent variables (Fig. 3). The risk factors affecting the progression of localized gingivitis to generalized gingivitis were dental calculus, large amount dental plaque, crowded teeth, bleeding teeth during brushing that occurred sometimes or was frequent, and large amount dental plaque is a risk factor for generalized gingivitis.

## Discussion

This study found a high prevalence of gingivitis in children aged 6–12 years in Jinzhou, China; however, in terms of severity, the prevalence of generalized gingivitis, which is less severe, was only 4.24%, with the majority of gingivitis being localized. Generalized gingivitis has a relatively low morbidity, with the majority of localized gingivitis. In a 2021 survey conducted in Guangdong Province, China, the prevalence rates of localized gingivitis and generalized gingivitis in 12-year-old children were 21.8% and 5.4%, respectively [17]. Both studies used the same diagnostic criteria for gingivitis that was used in this study; however, the gingival bleeding index used for the examination of gingival bleeding was not the same as the examination criteria (GI) used in this study. Therefore, the findings cannot be compared. It is difficult to compare the results of various studies because there is no clear-cut method for gingivitis screening. To study the prevalence and severity of gingivitis in children more comprehensively and systematically, we examined all teeth in our survey and defined and graded gingivitis using the GI.

Regarding the distribution of gingivitis within the mouth, the study has found that the gingiva of anterior teeth and their gingival papillae are more susceptible to the severity of gingivitis than that observed with the posterior teeth [1]. Addy et al. [18] explained that the possible reason for this is that cuspids and premolars are easier to clean, which may also be the reason why lateral incisors and molars are more likely to suffer from gingivitis. We found higher scores on the right side relative to the left side in all dental positions of the mouth, and similar results were obtained in a survey of gingivitis in primary school children in Bucharest, Romania, which suggested that gingivitis might be related to habitual hand brushing [19]. However, it has also been shown that there is no difference in the occurrence of gingivitis between left-handed and right-handed brushing [20]. Further longitudinal studies are required to confirm the association between handedness and gingivitis.

Some epidemiological reports have shown that gingivitis is more common in males than in females in the following two ways [8, 17, 19]. Firstly, females have better oral hygiene than that of males. On the other hand, studies have shown that sex hormones play an important role in influencing the progression of periodontal tissue disease [5]. In clinical trials, patients with sufficient estrogen have more dental plaque than those with insufficient estrogen, which, however, will not lead to,an increased morbidity of gingivitis [21]. Some studies have suggested that sex is a risk factor for gingivitis [19, 22]; however, this result was not found in our survey (*P* > 0.05). The *p*ossible causes are that peak gingivitis occurs in girls aged 11–13 years and in boys aged 13–14 years [23], and the current survey recruited respondents from different age ranges. The present investigation found that the occurrence of gingivitis was associated with increasing age, which is consistent with other experimental findings. Therefore, compared to older children, younger children have better periodontal health [22]. The prevalence of generalized gingivitis increased slightly with age but did not differ significantly in the one-way logistic regression analysis.

Medium amountand large amount dental plaque and dental calculus are risk factors for the development of gingivitis, which is consistent with findings from national and international studies [8, 17, 24]. Bacterial biofilm that forms a dental plaque is an important factor for triggering gingivitis, while calculus exists in the form of calcified biofilm and bacterial deposits in mineralized material [25]. Plaque accumulates more rapidly around inflamed gums than around gums without inflammation, and the plaque biofilm reacts immunologically with the host to produce an inflammatory infiltrate in the gingival tissue, leading to the development of gingivitis [26]. The extent of gingivitis increases as the amount and location of plaque buildup increase, further confirming the association of large amount plaque with generalized gingivitis. Once the plaque biofilm is removed, changes in periodontal tissue are completely reversible [11]. In addition, plaque is an indicator of poor oral hygiene; therefore, a good oral hygiene environment is important for the health of periodontal tissues.

Deep overjet and crowded teeth are closely related to the incidence and severity of gingivitis. Moreover,the occurrence of deep overjet can aggravate gingivitis.A previous study showed increased coverage and inflammation associated with lack of lip sealing, which causes dry environment and is detrimental to the health of gingival tissue [27]. Our study findings are consistent with that obtained in the previous study [27]. However, Thiam et al. showed that deep overjet was significantly associated with clinical attachment loss and the development of periodontal pockets but not with gingival inflammation and plaque accumulation [28]. Crowded teeth can damage the shape of the gums and alveolar bone and change the shape of the gingival papillae, further leading to thinning of the bone septum and reduction in blood vessels and cancellous bone, which makes the periodontal tissue less resistant to microbial attack [29]. There is evidence of a direct relationship between the number of contact areas overlapped by tooth displacement and the number of red, bleeding areas in the gums. There was also a significant correlation between dental crowding and the amount of plaque, with irregularities in tooth alignment, leading to an increase in plaque area on adjacent surfaces [28, 30]. The correlation between dental crowding and generalized gingivitis was further examined. Dental crowding is a risk factor for the development of gingivitis, but plaque control is key to controlling the development of gingivitis.

In terms of oral health behaviors and health awareness, we found that poor cleaning habits and poor health awareness increased the prevalence of gingivitis. Higher prevalence of gingivitis was observed in patients who have not flossed or brushing time is less than three minutes. Regular interdental and frequent dental cleaning are effective in reducing plaque and dental calculus, thereby reducing the incidence and severity of gingivitis [31]. Although toothbrushes alone have less impact on reducing plaque dental calculus and gingivitis, its combination with interdental cleaning maximizes the oral environment [32]. Occasional or frequent bleeding from tooth brushing is an important risk factor for the development and severity of gingivitis, indicating that this group is not aware that bleeding gums are associated with gingivitis. Self assessment based on the degree of toothbrushing bleeding is considered as an effective method to monitor gingival health [33]. Raising periodontal awareness in the population and reducing the prevalence and severity of gingivitis through individual gingival health monitoring. Therefore, there is a need to further enhance oral hygiene awareness and education regarding periodontal tissue care. Unsurprisingly, not visiting a dentist within a year is a risk factor for the onset and exacerbation of gingivitis. Therefore, it is essential to receive professional oral hygiene advice and develop good awareness of health care to reduce the incidence of gingivitis. When personal oral hygiene skills are poor, receiving guidance from a professional dentist before performing oral cleaning can complement the knowledge of oral health care and better improve oral hygiene [34]. 

Compared with similar studies, this study has the advantage of using a larger sample size to assess the current gingival health. In contrast, there are relatively few research on children's gingival health in China. Although studies have been conducted in other regions, it does not represent the incidence in Northeast China. However, this study still has potential limitations. Cross-sectional studies could only make hypotheses, but failed to explain the causal relationship between gingivitis and risk factors. Therefore the identified associations cannot be considered causal,which still need to be further proved in the longitudinal studies. We failed to perform deeper examination (x-rays, etc.) on children with gingivitis due to space limitations. This study will contribute to the epidemiological study of childer with gingivitis,,so as to provide a reference for the oral health care planning for school-age children in this region.

## Conclusions

Our findings showed shown that dental calculus, large amount dental plaque, poor oral health behavior, and oral health awareness are associated with the prevalence of gingivitis and that maintaining children’s oral health requires professional guidance and regular preventive care.

## Data Availability

The datasets generated and/or analysed during the current study are not publicly available due to privacy concerns but are available from the corresponding author on reasonable request.

## Abbreviations

BMI: Body mass index
CPITN: Community periodontal index
EFP/AAP: American Academy of Periodontology/European Federation of Periodontology
AL: Attachment loss
PD: Periodontal probing depth
GI: Gingival index
WHO: World Health Organization
OR: Odds ratio
CI: Confidence intervals
